# Automated quantification of atrophy and acute ischemic volume for outcome prediction in endovascular thrombectomy

**DOI:** 10.3389/fneur.2022.1056532

**Published:** 2022-12-15

**Authors:** Balázs Kis, Ain A. Neuhaus, George Harston, Olivier Joly, Davide Carone, Stephen Gerry, Zoltán Chadaide, András Pánczél, Eszter Czifrus, Viktória Csike, Ágnes Surányi, István Szikora, Loránd Erőss

**Affiliations:** ^1^National Institute of Mental Health, Neurology, and Neurosurgery (NIMNN), Budapest, Hungary; ^2^Oxford University Hospitals NHS Foundation Trust, Oxford, United Kingdom; ^3^Radcliffe Department of Medicine, University of Oxford, Oxford, United Kingdom; ^4^Brainomix Ltd., Oxford, United Kingdom; ^5^Centre for Statistics in Medicine, Nuffield Department of Orthopedics, Rheumatology and Musculoskeletal Sciences, University of Oxford, Oxford, United Kingdom; ^6^Faculty of Medicine, Semmelweis University, Budapest, Hungary

**Keywords:** stroke, endovascular thrombectomy, thrombolysis, neuroimaging, neuroradiology, artificial intelligence, machine learning

## Abstract

**Background:**

Short- and long-term outcomes from endovascular thrombectomy (EVT) for large vessel occlusion stroke remain variable. Numerous relevant predictors have been identified, including severity of neurological deficits, age, and imaging features. The latter is typically defined as acute changes (most commonly Alberta Stroke Programme Early CT Score, ASPECTS, at presentation), but there is little information on the impact of imaging assessment of premorbid brain health as a determinant of outcome.

**Aims:**

To examine the impact of automated measures of stroke severity and underlying brain frailty on short- and long-term outcomes in acute stroke treated with EVT.

**Methods:**

In 215 patients with anterior circulation stroke, who subsequently underwent EVT, automated analysis of presenting non-contrast CT scans was used to determine acute ischemic volume (AIV) and e-ASPECTS as markers of stroke severity, and cerebral atrophy as a marker of brain frailty. Univariate and multivariate logistic regression were used to identify significant predictors of NIHSS improvement, modified Rankin scale (mRS) at 90 and 30 days, mortality at 90 days and symptomatic intracranial hemorrhage (sICH) following successful EVT.

**Results:**

For long-term outcome, atrophy and presenting NIHSS were significant predictors of mRS 0–2 and death at 90 days, whereas age did not reach significance in multivariate analysis. Conversely, for short-term NIHSS improvement, AIV and age were significant predictors, unlike presenting NIHSS. The interaction between age and NIHSS was similar to the interaction of AIV and atrophy for mRS 0–2 at 90 days.

**Conclusion:**

Combinations of automated software-based imaging analysis and clinical data can be useful for predicting short-term neurological outcome and may improve long-term prognostication in EVT. These results provide a basis for future development of predictive tools built into decision-aiding software in stroke.

## Introduction

Outcomes in endovascular thrombectomy (EVT) for emergent large vessel occlusion (ELVO) stroke remain variable: only 46% of patients treated with EVT in early time-window trials achieved functional independence (modified Rankin scale, mRS 0–2) (1). In real-world registry studies, the overall functional independence has been reported to be as low as 23% after EVT (2). As such, improved patient selection criteria are required to maximize cost-effectiveness while minimizing unnecessary procedure-related risks.

Currently, identifying suitable candidates relies on clinical features (presenting severity on the National Institutes of Health Stroke Scale, NIHSS; baseline mRS), time from stroke onset (3) and imaging findings, including infarct volume (4), Alberta Stroke Programme Early CT Score (ASPECTS) (5) and volume of ischemic tissue relative to infarct (6). However, these radiological features all represent acute changes. There is increasing evidence that imaging biomarkers unrelated to the index stroke, including atrophy as an indicator of brain frailty, have a significant impact on clinical outcome (7, 8). However, there is no consensus on the best integrative approach to use these factors for long-term prognosis and poor understanding of how they interact. Furthermore, manual quantification of atrophy using visual analog scales can be subjective (9) while automated quantification usually requires separate software, which would complicate reporting workflow in the acute setting.

In this study, we sought to use machine learning based automated image analysis of routine non-contrast CT (NCCT) brain imaging, in conjunction with clinical variables, to improve prognostication for short-term neurological improvement (changes in NIHSS), long-term functional outcomes (mRS at 30 and 90 days), mortality and symptomatic intracranial hemorrhage. Our hypothesis was that automated image analysis of routine NCCT imaging markers of both stroke severity and brain frailty would be significant predictors of short- and long-term outcome alone following successful EVT.

## Methods

### Study design

This retrospective study was performed at the National Institute of Mental Health, Neurology and Neurosurgery (NIMNN) in Budapest, Hungary. From 1 January 2017 to 31 December 2019, we included patients receiving endovascular thrombectomy for acute ischemic stroke. All treatment decisions were made based on clinical criteria, as indicated according to first line international (3) and local guidelines. Further inclusion criteria included: age ≥18, causative middle cerebral artery M1 segment occlusion, onset to groin puncture ≤6 h, NIHSS score ≥ 6 at presentation, premorbid mRS state ≤1, ASPECTS ≥ 6, and successful recanalization (TICI ≥ 2b).

Since more than 90% of the patients are transferred from primary stroke centers to NIMNN for EVT, an NCCT scan was repeated on arrival for final decision-making regarding EVT. These preprocedural scans were defined as baseline imaging and processed using e-Stroke software (version 10; Brainomix, UK), a machine learning based decision aid tool for acute ischemic stroke, which has been validated against manual analysis by neuroradiologists in previous studies (10, 11). e-Stroke was used to estimate acute ischemic volume (AIV), automated e-ASPECTS, and atrophy (defined based on CSF volume in the lateral ventricles and surrounding brain parenchyma, relative to parenchymal volume). Patients with missing or inadequate baseline imaging were excluded from analyses.

We further collected patient demographics, baseline imaging features (including radiologist ASPECTS scores), treatment times (onset to groin, groin to recanalization, NIHSS, whether the patient received intravenous thrombolysis, TICI scores, short-term neurological outcome (NIHSS improvement at discharge or 7 days), longer term functional outcome (mRS at 30 and 90 days) and whether the patient suffered symptomatic intracranial hemorrhage [defined as European Cooperative Acute Stroke Study (ECASS) parenchymal hematoma 1 and 2 categories].

### Data analysis

Data were collected into a database as part of clinical care and therefore unblinded. Patients were invited for follow-up clinic appointments where mRS was determined by the reviewing physician; for patients who did not attend appointments, trained abstractors collected mRS over the phone. Statistical analyses were performed in R version 4.1.1 (12). Primary outcomes were considered as early, defined as dichotomized NIHSS improvement at discharge (reduction of NIHSS of ≥4 points in patients with a presenting NIHSS ≥4), and late, defined as dichotomized functional outcome at 90 days (with mRS 0–2 considered as good outcome). Secondary outcomes included mRS 0–2 at 30 days, intracranial hemorrhage, and mortality at 90 days. Univariate logistic regression was performed for manually selected variables. Multivariate logistic regression was performed with our four primary predictors of interest (presenting NIHSS and AIV as clinical and radiological indicators of acute severity, and age and atrophy as clinical and radiological indicators of brain frailty), alongside variables that were significant in univariate analyses (*p* < 0.05). As AIV and e-ASPECTS were highly collinear, the latter was not included in multivariate models even if significant in univariate analysis. Cases with missing outcome data were excluded from analysis for that model. Descriptive analysis was undertaken to explore the interaction between stroke severity and brain frailty on outcome, using both clinical and imaging biomarkers.

## Results

**Figure 1:**
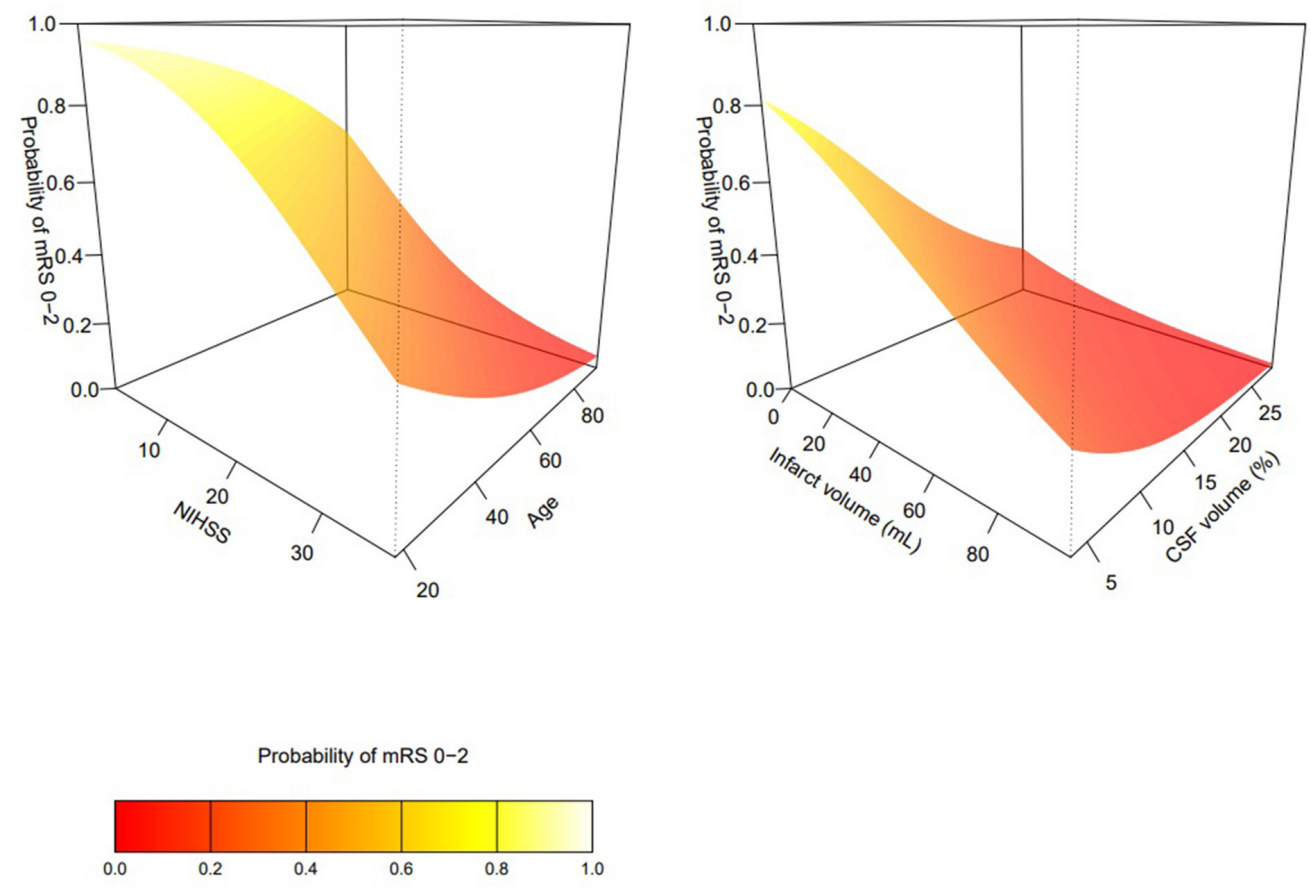
Interactions between age and NIHSS **(left)**, and infarct volume and atrophy **(right)** in predicting mRS 0–2 at 90 days.

The study population included 215 patients; demographic details and procedural characteristics are displayed in Table 1. Overall, 104 patients (48.4%) achieved mRS 0–2 at 90 days. The significant univariate and multivariate predictors of achieving mRS 0–2 are summarized in Table 2. Briefly, univariate analyses showed that likelihood of good outcome was significantly associated with age, NIHSS on admission, NCCT AIV, e-ASPECTS, atrophy and TICI status. When adjusted in multivariate regression, however, only atrophy, NIHSS on admission and TICI 2C/3 retained statistical significance. The cumulative effect of age and NIHSS, and separately infarct volume, and atrophy on functional outcome are shown in Figure 1. The pattern of interaction between stroke severity and surrogates of baseline frailty showed a similar relationship when assessed using both clinical and imaging characteristics.

**Table 1:** Demographic details of the study cohort.

**Age (years)**	**67.6 (SD 14.1)**
**Female**	**128 (59.5%)**
**Presenting features**	
**Left-sided occlusion**	**103 (47.9%)**
**NIHSS on admission**	**15 (IQR 12–19)**
**Manual ASPECTS on admission**	**8 (IQR 7–8)**
**e-ASPECTS on admission**	**8 (IQR 8–9)**
**NCCT infarct volume on admission (mL)**	**17.5 (SD 15.6)**
**Atrophy on admission (% brain volume)**	**11.48 (SD 4.64)**
**Comorbidities**	
**Atrial fibrillation**	**91 (42.3%)**
**Hypertension**	**163 (75.8%)**
**Diabetes mellitus**	**48 (22.3%)**
**Ischaemic heart disease**	**34 (15.8%)**
**Peripheral arterial disease**	**27 (12.6%)**
**Prior stroke**	**26 (12.1%)**
**Procedural features**	
**Onset to recanalization (min)**	**287.2 (SD 68.3)**
**Onset to groin (min)**	**251 (SD 65.1)**
**Door to groin (min)**	**49.8 (SD 26.4)**
**Groin to recanalization (min)**	**36.7 (SD 22.2)**
**TICI 2B**	**71 (33.0%)**
**TICI 2C**	**32 (14.9%)**
**TICI 3**	**112 (52.1%)**
**First pass recanalization**	**122 (56.7%)**
**IV thrombolysis**	**130 (60.5%)**

**Table 2:** Univariate and multivariate predictors of mRS 0–2 at 90 days.

	**mRS 0-2 at 90 days**		
**Predictors**	**Odds Ratios**	**CI**	***p***
**Univariate**			
(Intercept)	13.92	3.38–63.91	**<0.001**
Age	0.96	0.94–0.98	**<0.001**
NIHSS at admission	0.89	0.84–0.94	**<0.001**
AIV	0.98	0.96–0.99	**0.017**
Atrophy	0.88	0.82–0.94	**<0.001**
TICI 2C/3	2.05	1.15–3.70	**0.016**
e-ASPECTS	1.26	1.04–1.56	**0.021**
IV thrombolysis	1.28	0.74–2.21	0.385
Onset to recanalisation	1	0.99–1.00	0.066
**Multivariate**			
(Intercept)	44.54	7.78–299.35	**<0.001**
Age	0.98	0.95–1.01	0.113
NIHSS at admission	0.9	0.84–0.95	**0.001**
AIV	0.98	0.96–1.00	0.127
Atrophy	0.91	0.83–0.99	**0.038**
TICI 2C/3	3	1.55–5.99	**0.001**

The bold values indicate the statistical significance of p < 0.05.

Using the same approach, we then looked at early neurological outcomes following thrombectomy. NIHSS improvement data were available in 203 patients, with six patients excluded due to missing follow up NIHSS and six patients excluded due to baseline severity of NIHSS < 4. NIHSS improvement was achieved by 162 patients (79.8%), albeit with a wide range of absolute NIHSS values at discharge (range 0–30, IQR 2–11). The univariate and multivariate predictors for NIHSS improvement are summarized in Table 3. In univariate analyses, only AIV and onset to recanalization time were associated with NIHSS improvement. After adjustment in multivariate analysis, age, AIV and onset to recanalization demonstrated statistical significance.

**Table 3:** Univariate and multivariate predictors of NIHSS improvement.

	**NIHSS improvement**		
**Predictors**	**Odds Ratios**	**CI**	***p***
**Univariate**			
Age	0.98	0.95–1.01	0.141
NIHSS at admission	1.02	0.95–1.09	0.637
AIV	0.97	0.94–0.99	0.001
Atrophy	1.01	0.94–1.10	0.792
TICI 2C/3	1.97	0.97–3.99	0.059
e-ASPECTS	1.20	0.95–1.50	0.116
IV thrombolysis	1.43	0.71–2.86	0.311
Onset to recanalisation	0.99	0.98–1.00	0.001
**Multivariate**			
(Intercept)	3983.64	140.65–178884.09	<0.001
Age	0.94	0.90–0.98	**0.004**
NIHSS at admission	1.08	1.00–1.18	0.061
AIV	0.95	0.92–0.97	**<0.001**
Atrophy	1.12	0.99–1.28	0.082
Onset to recanalisation	0.99	0.98–0.99	**<0.001**

The bold values indicate the statistical significance of p < 0.05.

We performed the same analyses for mRS 0–2 at 30 days, achieved by 90 patients (41.9%). Univariate and multivariate predictors are displayed in Supplementary Table 1. In univariate analysis, age, AIV, NIHSS, atrophy and TICI 2C/3 status were all associated with functional independence at 30 days, similarly to the 90-day mRS; in the adjusted analysis, only age, AIV and TICI retained significance. At 90 days, 65 patients (30.2%) had died. The univariate and multivariate analyses for mortality are in Supplementary Table 2; age, admission NIHSS and atrophy were associated with death in univariate analysis, while only NIHSS and atrophy demonstrated statistical significance in multivariate analysis. We also attempted to examine post-procedural sICH, but this was limited by sample size as only 12 patients developed parenchymal hemorrhage type 1 or 2. NIHSS on admission was the only variable to show significant association to sICH in both univariate and multivariate analysis (Supplementary Table 3).

## Discussion

Imaging biomarkers automatically derived from CT imaging routinely acquired at presentation provided information on both the acute injury sustained (acute infarct volume) and on the premorbid condition of the brain (brain atrophy). Both clinical and imaging markers of stroke severity, i.e., presenting NIHSS and acute infarct volume, offer potentially useful information when predicting neurological improvement. Furthermore, there is additional information to be gained from both clinical and imaging markers of brain frailty when predicting early outcomes. The interactions between markers of stroke severity and surrogates of frailty appear similar whether clinical or radiological variables are used.

Atrophy was more strongly associated with long-term outcome compared to age. Compared to age, atrophy likely provides a better estimate of brain frailty, a key determinant of vulnerability to injury (13). Metrics capturing biological age, as opposed to calendar age, might also better inform of the capacity of an individual to recover following stroke as they may better represent the brain neurological reserve and the ability to compensate for an infarct of given volume following stroke.

While long term outcome is influenced by multiple factors, such as post EVT care, rehabilitation, and comorbid factors, the early neurological outcome might be argued to be more sensitive to the impact of factors related to the index stroke severity and degree of procedural success. It is unclear why age rather than atrophy demonstrated this association in these results; hypothetically, non-neurological factors such as musculoskeletal comorbidity may contribute to the potential for early neurological compensation which is more likely to be captured by age than a neurologically specific biomarker such as atrophy.

The influence of the individual biomarkers used in this study is broadly consistent with previous observations. e-ASPECTS, which is derived from the automated AIV, has been demonstrated to predict mRS at 90 days in other cohorts (14). Similarly, automated atrophy quantification strongly predicts mRS, with an odds ratio of 0.65 per 5% increase in intracranial cerebrospinal fluid volume (15). A previous machine learning study of NCCT and CTA features using e-Stroke found that age, baseline NIHSS, occlusion side, atrophy and e-ASPECTS were the best predictors for mRS at 90 days, in keeping with the results shown here (16). The clinical variables used in this study—age and presenting NIHSS—have previously been used as a prognostic score, which was also an independent predictor of outcome in EVT (17). Other studies have also found significant predictive effects from age and NIHSS at presentation (18), and there is a combined effect between NIHSS and age in agreement with the data presented here (19). Procedurally, there is a strong correlation between treatment times and outcome, particularly in the early window (2). Excellent reperfusion (TICI 2C/3) is also highly predictive or mRS at 3 months, especially when this is achieved during the first pass (20).

There is comparatively less data on predictors of early neurological improvement which is more directly linked to the immediate impact of an intervention, and most of these focus on the earliest time windows. One study identified age, blood glucose, TICI, baseline ASPECTS and the presence of sICH as predictors for early neurological recovery (21). Others have looked at predictive factors for failure to neurologically improve, and identified variables including premorbid mRS, hyperglycemia, longer treatment times, lack of tPA bridging, and involvement of motor cortex and internal capsule in the infarct (22). Separately, there is considerable literature suggesting the utility of early neurological improvement itself as a long-term prognostic factor. In a large cohort study, absolute NIHSS at 24 h was the best predictor of mRS at 90 days, although NIHSS improvement was also strongly correlated with long-term outcome (23). Notably, in our cohort AIV was a better predictor of early neurological improvement than atrophy, illustrating the differential impact of acute and chronic brain changes to short- and long-term recovery.

Our study has limitations. First, although the group sizes of those achieving mRS 0–2 and 3–6 were similar, the overall sample size was relatively small at 215 patients. In addition, the intracerebral hemorrhage analyses were underpowered as there were only 12 cases in the cohort. Second, this was a single-center retrospective cohort study. Validation in prospective cohorts is required. Third, our hypotheses and choices of variables were clinically driven, and we cannot exclude that there are other imaging parameters that may further improve performance but were not captured in this dataset.

Overall, these data support the use of automated imaging analysis for improving the prediction of neurological recovery following EVT and demonstrate the additional information that can be captured from simple NCCT imaging. The results show the interaction between markers of stroke severity and the brain frailty of the individual and suggest an opportunity to refine estimates of an individual's capacity to recover beyond that of their chronological age. If validated in prospective cohorts, this may provide a useful adjunct tool for prognostication in large vessel occlusion ischemic stroke.

## Data availability statement

The raw data supporting the conclusions of this article will be made available by the authors, without undue reservation.

## Ethics statement

The studies involving human participants were reviewed and approved by National Institute of Mental Health, Neurology and Neurosurgery (NIMNN), Budapest, Hungary. Written informed consent for participation was not required for this study in accordance with the national legislation and the institutional requirements.

## Author contributions

BK, ZC, AP, EC, VC, ÁS, IS, and LE designed the study and collected data. BK, AN, and SG performed data analysis. GH and OJ provided essential data analysis tools. AN, GH, and DC drafted the manuscript. All authors were involved with manuscript revisions and contributed to the article and approved the submitted version.

## References

[1] Menon BK van Zwam WH Dippel DWJ Mitchell PJ Demchuk AM. Endovascular thrombectomy after large-vessel ischaemic stroke: a meta-analysis of individual patient data from five randomised trials. The Lancet. (2016) 387:1723–31. 10.1016/S0140-6736(16)00163-X 26898852

[2] Jahan R Saver JL Schwamm LH Fonarow GC Liang L Matsouaka RA. Association between time to treatment with endovascular reperfusion therapy and outcomes in patients with acute ischemic stroke treated in clinical practice. JAMA. (2019) 322:252–63. 10.1001/jama.2019.8286 31310296 PMC6635908

[3] Powers WJ Rabinstein AA Ackerson T Adeoye OM Bambakidis NC Becker K. Guidelines for the early management of patients with acute ischemic stroke: 2019 update to the 2018 guidelines for the early management of acute ischemic stroke: a guideline for healthcare professionals from the american heart association/american stroke association. Stroke. (2019) 50:e344–418. 10.1161/STR.0000000000000211 31662037

[4] Boers AMM Jansen IGH Beenen LFM Devlin TG San Roman L Heo JH. Association of follow-up infarct volume with functional outcome in acute ischemic stroke: a pooled analysis of seven randomized trials. J Neurointerv Surg. (2018) 10:1137–42. 10.1136/neurintsurg-2017-013724 29627794

[5] Cagnazzo F Derraz I Dargazanli C Lefevre PH Gascou G Riquelme C. Mechanical thrombectomy in patients with acute ischemic stroke and ASPECTS 6: a meta-analysis. J Neurointerv Surg. (2020) 12:350–5. 10.1136/neurintsurg-2019-015237 31401563

[6] Albers GW Marks MP Kemp S Christensen S Tsai JP Ortega-Gutierrez S. Thrombectomy for Stroke at 6 to 16 hours with selection by perfusion imaging. N Engl J Med. (2018) 378:708–18. 10.1056/NEJMoa1713973 29364767 PMC6590673

[7] Lauksio I Lindstrom I Khan N Sillanpaa N Hernesniemi J Oksala N. Brain atrophy predicts mortality after mechanical thrombectomy of proximal anterior circulation occlusion. J Neurointerv Surg. (2021) 13:415–20. 10.1136/neurintsurg-2020-016168 32620574

[8] Pedraza MI de Lera M Bos D Calleja AI Cortijo E Gomez-Vicente B. Brain atrophy and the risk of futile endovascular reperfusion in acute ischemic stroke. Stroke. (2020) 51:1514–21. 10.1161/STROKEAHA.119.028511 32188368

[9] Harper L Barkhof F Fox NC Schott JM. Using visual rating to diagnose dementia: a critical evaluation of MRI atrophy scales. J Neurol Neurosurg Psychiatry. (2015) 86:1225–33. 10.1136/jnnp-2014-310090 25872513

[10] Sundaram VK Goldstein J Wheelwright D Aggarwal A Pawha PS Doshi A. Automated ASPECTS in acute ischemic stroke: a comparative analysis with CT perfusion. AJNR Am J Neuroradiol. (2019) 40:2033–8. 10.3174/ajnr.A6303 31727750 PMC6975365

[11] Wolff L Berkhemer OA van Es A van Zwam WH Dippel DWJ Majoie C. Validation of automated alberta stroke program early ct score (ASPECTS) software for detection of early ischemic changes on non-contrast brain CT scans. Neuroradiology. (2021) 63:491–8. 10.1007/s00234-020-02533-6 32857212 PMC7966210

[12] R Core Team. R: A Language and Environment for Statistical Computing. Vienna: R Foundation for Statistical Computing (2021).

[13] Evans NR Todd OM Minhas JS Fearon P Harston GW Mant J. Frailty and cerebrovascular disease: concepts and clinical implications for stroke medicine. Int J Stroke. (2022) 17:251–9. 10.1177/17474930211034331 34282986 PMC8864332

[14] Pfaff J Herweh C Schieber S Schonenberger S Bosel J Ringleb PA. e-ASPECTS correlates with and is predictive of outcome after mechanical thrombectomy. AJNR Am J Neuroradiol. (2017) 38:1594–9. 10.3174/ajnr.A5236 28596195 PMC7960437

[15] Diprose WK Diprose JP Wang MTM Tarr GP McFetridge A Barber PA. Automated measurement of cerebral atrophy and outcome in endovascular thrombectomy. Stroke. (2019) 50:3636–8. 10.1161/STROKEAHA.119.027120 31558139

[16] Jabal MS Joly O Kallmes D Harston G Rabinstein A Huynh T. Interpretable machine learning modeling for ischemic stroke outcome prediction. Front Neurol. (2022) 13:884693. 10.3389/fneur.2022.884693 35665041 PMC9160988

[17] Almekhlafi MA Davalos A Bonafe A Chapot R Gralla J Pereira VM. Impact of age and baseline NIHSS scores on clinical outcomes in the mechanical thrombectomy using solitaire FR in acute ischemic stroke study. AJNR Am J Neuroradiol. (2014) 35:1337–40. 10.3174/ajnr.A3855 24557701 PMC7966577

[18] Reddy ST Friedman E Wu TC Arevalo O Zhang J Rahbar MH. Rapid infarct progression in anterior circulation large vessel occlusion ischemic stroke patients during inter-facility transfer. J Stroke Cerebrovasc Dis. (2020) 29:105308. 10.1016/j.jstrokecerebrovasdis.2020.105308 32992188 PMC7686251

[19] Ospel JM Brown S Kappelhof M van Zwam W Jovin T Roy D. Comparing the prognostic impact of age and baseline national institutes of health stroke scale in acute stroke due to large vessel occlusion. Stroke. (2021) 52:2839–45. 10.1161/STROKEAHA.120.032364 34233465

[20] Yoo AJ Soomro J Andersson T Saver JL Ribo M Bozorgchami H. Benchmarking the extent and speed of reperfusion: first pass tici 2c-3 is a preferred endovascular reperfusion endpoint. Front Neurol. (2021) 12:669934. 10.3389/fneur.2021.669934 34046008 PMC8144635

[21] Zhang X Peng M Feng C Wang H Gong P Jiang T. Nomogram predicting early neurological improvement in ischaemic stroke patients treated with endovascular thrombectomy. Eur J Neurol. (2021) 28:152–60. 10.1111/ene.14510 32897575

[22] Weyland CS Mokli Y Vey JA Kieser M Herweh C Schonenberger S. Predictors for failure of early neurological improvement after successful thrombectomy in the anterior circulation. Stroke. (2021) 52:1291–8. 10.1161/STROKEAHA.120.030519 33626903

[23] Meyer L Broocks G Bechstein M Flottmann F Leischner H Brekenfeld C. Early clinical surrogates for outcome prediction after stroke thrombectomy in daily clinical practice. J Neurol Neurosurg Psychiatry. (2020) 91:1055–9. 10.1136/jnnp-2020-323742 32934109

