# Supplementary material for: Automated quantification of atrophy and acute ischemic volume for outcome prediction in endovascular thrombectomy
Source: Front Neurol. 2022 Dec 15;13:1056532. doi: 10.3389/fneur.2022.1056532 (PMC9797714; doi:10.3389/fneur.2022.1056532)
Supplement: Supplementary file 1 [file Data_Sheet_1.docx]

# Supplementary Table 1. mRS 0-2 at 30 days

| **Univariate** | **mRS 0-2 at 30 days** | | |
| --- | --- | --- | --- |
| *Predictors* | *Odds Ratios* | *CI* | *p* |
| Age | 0.96 | 0.94 – 0.98 | **<0.001** |
| NIHSS at admission | 0.87 | 0.82 – 0.92 | **<0.001** |
| AIV | 0.98 | 0.95 – 0.99 | **0.018** |
| Atrophy | 0.92 | 0.86 – 0.98 | **0.008** |
| TICI 2C/3 | 2.41 | 1.32 – 4.52 | **0.005** |
| e-ASPECTS | 1.26 | 1.03 – 1.56 | **0.029** |
| IV thrombolysis | 1.45 | 0.83 – 2.55 | 0.196 |
| Onset to recanalisation | 1.00 | 0.99 – 1.00 | 0.177 |

| **Multivariate** | **mRS 0-2 at 30 days** | | |
| --- | --- | --- | --- |
| *Predictors* | *Odds Ratios* | *CI* | *p* |
| (Intercept) | 34.73 | 6.06 – 229.84 | **<0.001** |
| Age | 0.97 | 0.94 – 1.00 | **0.043** |
| NIHSS at admission | 0.87 | 0.81 – 0.93 | **<0.001** |
| AIV | 0.98 | 0.96 – 1.01 | 0.219 |
| Atrophy | 0.96 | 0.88 – 1.05 | 0.403 |
| TICI 2C/3 | 3.88 | 1.94 – 8.18 | **<0.001** |

# Supplementary Table 2. Mortality at 90 days

| **Univariate** | **Mortality at 90 days** | | |
| --- | --- | --- | --- |
| *Predictors* | *Odds Ratios* | *CI* | *p* |
| Age | 1.05 | 1.02 – 1.08 | **<0.001** |
| NIHSS at admission | 1.12 | 1.05 – 1.18 | **<0.001** |
| AIV | 1.02 | 1.00 – 1.03 | 0.089 |
| Atrophy | 1.17 | 1.09 – 1.26 | **<0.001** |
| TICI 2C/3 | 0.95 | 0.51 – 1.77 | 0.866 |
| e-ASPECTS | 0.95 | 0.78 – 1.17 | 0.617 |
| IV thrombolysis | 0.68 | 0.37 – 1.22 | 0.192 |
| Onset to recanalisation | 1.00 | 1.00 – 1.01 | 0.289 |

| **Multivariate** | **Mortality at 90 days** | | |
| --- | --- | --- | --- |
| *Predictors* | *Odds Ratios* | *CI* | *p* |
| (Intercept) | 0.00 | 0.00 – 0.03 | **<0.001** |
| Age | 1.02 | 0.99 – 1.05 | 0.278 |
| NIHSS at admission | 1.11 | 1.04 – 1.18 | **0.001** |
| AIV | 1.01 | 0.99 – 1.03 | 0.304 |
| Atrophy | 1.15 | 1.05 – 1.26 | **0.002** |

# Supplementary Table 3. Haemorrhage

| **Univariate** | **Haemorrhage** | | |
| --- | --- | --- | --- |
| *Predictors* | *Odds Ratios* | *CI* | *p* |
| Age | 1.02 | 0.98 – 1.07 | 0.362 |
| NIHSS at admission | 1.13 | 1.02 – 1.26 | **0.020** |
| AIV | 1.01 | 0.97 – 1.04 | 0.514 |
| Atrophy | 1.08 | 0.96 – 1.20 | 0.162 |
| TICI 2C/3 | 0.67 | 0.21 – 2.35 | 0.515 |
| e-ASPECTS | 0.80 | 0.57 – 1.17 | 0.216 |
| IV thrombolysis | 0.91 | 0.28 – 3.17 | 0.877 |
| Onset to recanalisation | 1.01 | 1.00 – 1.02 | 0.163 |

| **Multivariate** | **Haemorrhage** | | |
| --- | --- | --- | --- |
| *Predictors* | *Odds Ratios* | *CI* | *p* |
| (Intercept) | 0.00 | 0.00 – 0.09 | **0.004** |
| Age | 1.00 | 0.94 – 1.06 | 0.969 |
| NIHSS at admission | 1.13 | 1.02 – 1.26 | **0.024** |
| AIV | 1.00 | 0.96 – 1.03 | 0.923 |
| Atrophy | 1.09 | 0.93 – 1.26 | 0.248 |
